# The effect of a prospective intervention program with automated monitoring of hand hygiene performance in long-term and acute-care units at a Veterans Affairs medical center

**DOI:** 10.1017/ice.2023.186

**Published:** 2024-02

**Authors:** W. Grant Starrett, James W. Arbogast, Albert E. Parker, Pamela T. Wagner, Susan E. Mahrer, Vanessa Christian, Barbara L. Lane, V. Lorraine Cheek, Gregory A. Robbins, John M. Boyce, Hari Polenakovik

**Affiliations:** 1 Dayton Veterans Affairs Medical Center, Dayton, Ohio, USA; 2 Division of Infectious Diseases, Department of Medicine, Wright State University, Dayton, Ohio, USA; 3 GOJO Industries, Akron, Ohio, USA; 4 Center for Biofilm Engineering, Montana State University, Bozeman, Montana, USA; 5 Department of Mathematical Sciences, Montana State University, Bozeman, Montana, USA; 6 JM Boyce Consulting, Middletown, Connecticut, USA

## Abstract

**Objective:**

To measure the impact of an automated hand hygiene monitoring system (AHHMS) and an intervention program of complementary strategies on hand hygiene (HH) performance in both acute-care and long-term care (LTC) units.

**Design:**

Prospective, nonrandomized, before-and-after intervention study.

**Setting:**

Single Veterans Affairs Medical Center (VAMC), with 2 acute-care units and 6 LTC units.

**Methods:**

An AHHMS that provides group HH performance rates was implemented on 8 units at a VAMC from March 2021 through April 2022. After a 4-week baseline period and 2.5-week washout period, the 52-week intervention period included multiple evidence-based components designed to improve HH compliance. Unit HH performance rates were expressed as the number of dispenses (events) divided by the number of patient room entries and exits (opportunities) × 100. Statistical analysis was performed with a Poisson general additive mixed model.

**Results:**

During the 4-week baseline period, the median HH performance rate was 18.6 (95% CI, 16.5–21.0) for all 8 units. During the intervention period, the median HH rate increased to 21.6 (95% CI, 19.1–24.4; *P* < .0001), and during the last 4 weeks of the intervention period (exactly 1 year after baseline), the 8 units exhibited a median HH rate of 25.1 (95% CI, 22.2–28.4; *P* < .0001). The median HH rate increased from 17.5 to 20.0 (*P* < .0001) in LTC units and from 22.9 to 27.2 (*P* < .0001) in acute-care units.

**Conclusions:**

The intervention was associated with increased HH performance rates for all units. The performance of acute-care units was consistently higher than LTC units, which have more visitors and more mobile veterans.

Emerging evidence indicates that effective implementation of an automated hand hygiene monitoring system (AHHMS) must be part of a multimodal hand hygiene (HH) program that includes complementary strategies.^1–3^ Few published studies have described in detail the intervention strategies used in conjunction with an AHHMS. Although national and international guidelines^4,5^ provide valuable resources for training, implementation, and monitoring of HH in healthcare settings, these guidelines do not yet address the effective use of AHHMS.

AHHMS is a quantitative approach to augment direct observation of healthcare worker (HCW) HH compliance, providing real-time feedback on the performance of HH.^6,7^ Multiple studies have shown that an AHHMS in combination with intervention efforts can have a positive impact on HH in acute-care units.^1,3,8,9^ Additionally, limited evidence suggests that the collaboration of healthcare patient-safety leadership and the AHHMS vendor can contribute to successfully turning HH intervention efforts into a sustained increase in HH compliance in acute-care units.^1,2^ However, little published evidence is available on the effectiveness of an AHHMS in long-term care (LTC) units, including nursing homes.^10^

The Dayton Veterans Affairs Medical Center (VAMC) includes an 80-bed acute-care hospital, 185-bed LTC facility, and a 91-bed domiciliary. To improve HH compliance and prevent healthcare-associated infections (HAIs), we conducted an 8-week pilot study utilizing the AHHMS on one of the LTC units in 2019. Results were encouraging, and the executive leadership team approved a permanent AHHMS installation in all acute-care and LTC units, which was accomplished in early 2021. We describe the effect of a designed program of serial complementary interventions on the HH rate in the first year of implementation.

## Methods

We analyzed data from a group-based AHHMS without badges (PURELL SMARTLINK Activity Monitoring System, GOJO Industries, Akron, OH) that uses battery-powered sensors on touch-free dispensers to record alcohol-based hand rub (ABHR) and soap dispensing events. Room sensors count patient room entries and exits by all individuals (eg, healthcare providers, patients, ancillary staff and visitors). Data are reported online by unit. This system offers group-level monitoring with 24/7 data collection and wireless transmission of data to provide an HH overview. Elements of this exact system have been described previously in detail.^11^ Unit HH performance rates are expressed as the number of dispensing events divided by the number of patient room entries and exits (opportunities) × 100. The AHHMS was implemented on 2 medical-surgical acute-care units and 6 LTC units at the Dayton VAMC from March 2021 through April 2022. A third acute-care unit—the intensive care unit—was excluded from this study because it underwent multiple construction changes and AHHMS installation efforts during this study period.

Baseline rates for each unit were established during a 4-week period immediately following AHHMS installation, and the 52-week intervention period commenced in April 2021, after a 2.5-week washout period. In addition to implementing the AHHMS, direct observation of HH compliance continued during the baseline and intervention periods performed by the Dayton VAMC infection prevention team (hereafter referred to as “VAMC investigators”). The VAMC and AHHMS vendor investigators collaboratively developed a “playbook” listing of complementary strategies based on previous experience and the body of evidence with AHHMS (Table 1). This tool listed all interventions, guided when to implement them, and defined the roles divided between the VAMC and AHHMS vendor investigators. We identified 4 phases of implementation: (1) explore and perform trials, (2) installation and education, (3) initial improvement, and (4) annual reassessment. These phases are further described in Table 1, and the intervention period we analyzed in this study encompassed phases 2 and 3.

**Table 1.** Automated Hand Hygiene Monitoring Systems (AHHMS) Intervention Checklist/Playbook Listing of Complementary Strategies by Phase of the AHHMS Implementation

Task or Milestone	Major Commitment From		Timing and Coaching Comment
	Healthcare	Vendor	
**Phase 1: Explore and perform trial**			∼3–6 months
Healthcare leader(s) engaged	√		Commitment to learn and desire to change is critical.
Trial installation, testing, analysis, decisions	√	√	
**Phase 2: Installation, validation, education**	Healthcare	Vendor	∼3–6 months
System installed		√	
System validation for functionality		√	
System planned path validation	√		
System behavioral path validation	√		
Conduct a “foot-traffic” assessment to roughly determine room entries plus exits from non-HCWs (% HCWs vs non-HCWs)^34^	√	√	
Educate via “train the trainer” sessions		√	
Unit managers inform and educate their unit	√		
Baseline hand hygiene performance rate established, but not reported		√	Set this over a 4-week period. No leaks of results during this period.
Baseline hand hygiene performance rate established and results communicated	√	√	
Goal setting (unit and/or individual)	√	√	
Initial HH improvement plans determined	√	√	
**Phase 3: Initial improvement—Complementary intervention strategies**	Healthcare	Vendor	∼6–24 months
Engaged, supportive nurse manager^35^	√	√	At the unit level
Initial HH goals and plans communicated	√	√	Done broadly and effectively
Unit-based champion assigned for all shifts (must include nights and weekends)^36^,^a^	√		Ideally not only the nurse manager
Unit-based data champion considered and/or assigned as appropriate^35^	√		Unit clerks could be a resource in making this happen.
Strong healthcare leader(s) engaged and encouraging improvement^1,3,8^	√		Leader(s) recognized by clinical staff is critical and motivating.
Identify and discuss barriers, optimize workflows and optimize dispenser placement^1^	√	√	
Decision on how to report results to units regularly (to whom, by whom, frequency)^1,3,8^,^b^	√	√	Style and/or frequency adapted to the healthcare facility plus unit culture
Weekly accountability calls and/or huddles^8,19^	√		All units represented is critical.
Discuss other hospital- and unit-based initiatives to promote change^3,8^	√	√	Such as Plan–Do–Check–Act, other hospitals, Quality best practices
Collaboration with unit leadership to establish ownership and engagement adapted to unit culture (“frontline ownership”)^37^	√	√	Reassess this a few months after baseline rollout
Critically review the facility hand hygiene policy; update and train as needed	√	√	
WHO HH self-assessment framework,^38^ review and consider application	√	√	Self-assessment with this tool may be better in ∼year 2.
Opportunistically bring broad visibility to colleagues (eg, grand rounds speaker)	√		
Opportunistically celebrate successes and leverage rewards and/or recognition	√		Potentially unit winners; rollout relatively slowly and thoughtfully
Optional: Improve direct observation practices			Such as real-time feedback on technique and/or missed moments
**Phase 4: Improvement efforts continued/adapted/optimized annually**	Healthcare	Vendor	Approximately annually thereafter
Annual assessment and program improvements determined	√	√	
Keep at it annually!	√	√	

Note. HCW, healthcare worker; HH, hand hygiene; WHO, World Health Organization.

a The role of HH champion is not just for auditing but also for being a “cheerleader.” The champion supporting the unit nurse manager may be rotated to another unit, but personnel that have experience in the unit and reflect the unit culture are preferable.

b Consider many different reporting options, including posting results on bulletin boards; monitors for unit leaders only (eg, inside the nurse station); monitors for display to anyone on the unit (HCWs, patients, and visitors); shift results for each unit reported daily to nurse managers and/or other designees (ideally to at least 2 people per unit); and results reported to hospital leadership team (as frequently as will be accepted and acted upon).

**Figure. 1.**
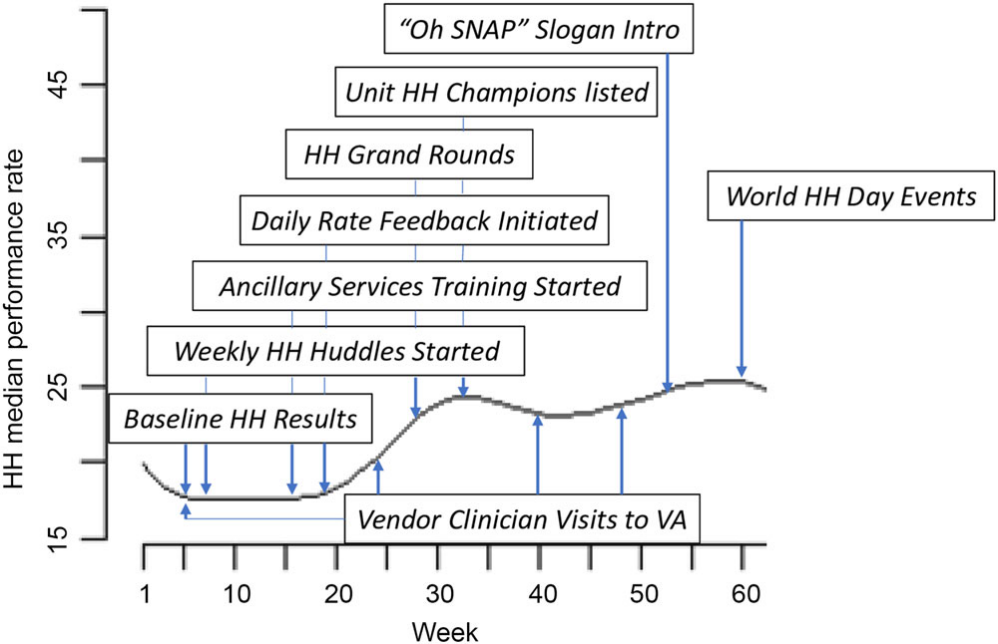
Dayton Veterans Affairs Medical Center combined hand hygiene rates and key events timeline.

Nurse managers from the 8 units were informed that the baseline period would last 4 weeks, during which time they received training in the use of the AHHMS. No training occurred during the following 2.5-week washout period, when baseline unit-specific performance rates were calculated. Baseline HH performance rates were subsequently provided to each unit’s nurse manager, and initial goals were set for each unit in cooperation with the VAMC and vendor investigators. A weekly HH “huddle” was scheduled between the nursing leadership and VAMC investigators beginning in week 7 (Fig. 1). In this huddle, the week’s rates for each unit were discussed, challenges and successes were shared, and future strategies were planned. Unit-specific rates were provided daily to nurse managers beginning in week 19, after the need for more frequent feedback was recognized. HH rate goals were raised for each unit as prior goals were met, utilizing interventions cumulatively implemented from the playbook as well as tips identified in the weekly huddles. Ancillary departments were trained in use of the AHHMS, and representatives of those performing high-frequency services were recruited for regular participation in the weekly huddle discussions. HH “champions” were chosen for each unit to assist the nurse managers, to take the initiative in tracking HH rates through the utilization of the online SMARTLINK tool, and to review vendor-supplied weekly data. These steps allowed them to identify actionable barriers and to provide ongoing education for their peers.

During the intervention period, the VAMC investigators critically reviewed and suggested a revision to the Dayton VAMC HH policy. Although it was not formally approved during that timeframe, the revision reflected principles used in training throughout the intervention period. These included a more detailed description of the proper use of ABHR,^4,5^ with a focus on coverage of thumbs and fingertips. The policy emphasized that an HCW performs HH before entering and upon leaving the patient room even if the HCW believed nothing was touched, and regardless of glove use. An exception was introduced from the World Health Organization (WHO) guidelines, allowing for a single HH event if leaving one patient room and immediately entering another.^5^ Our approach to calculating HH performance rates was unable to account for this possibility.

**Figure 2.**
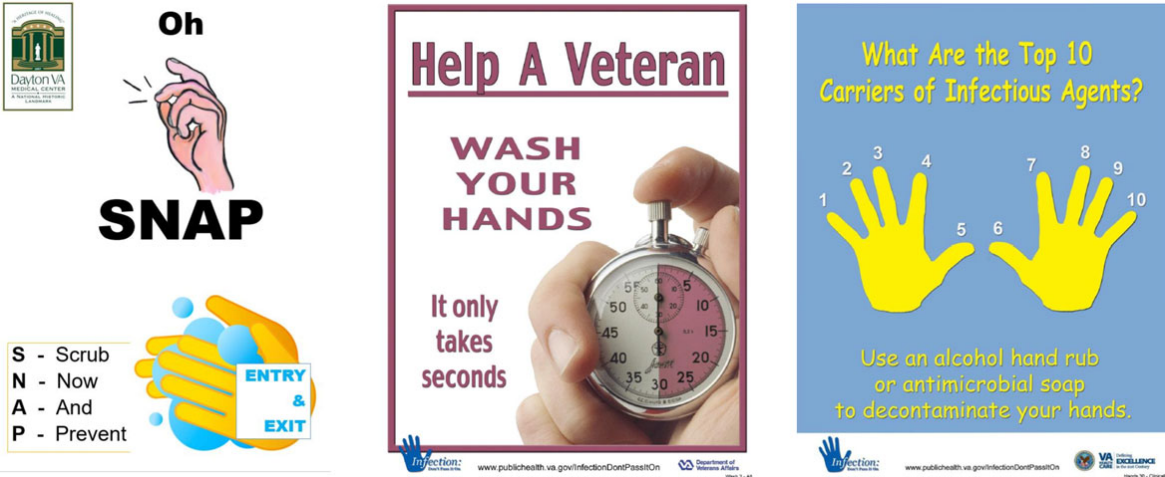
Slogan and signage examples.

Additional interventions included (1) a grand rounds focused on HH, which was delivered by a nationally recognized expert on the subject; (2) periodic appearances on the VAMC weekly “Fireside Chat” for employees to discuss the AHHMS and recognize the best-performing units, nurse managers and HH champions; and (3) development of signage and a new slogan (Fig. 2) to remind HCWs to perform HH. Finally, regular involvement by the vendor, including data analysis and periodic visits to the facility, were provided for focused clinician-based training and feedback.

Data were summarized by monthly HH performance rates equal to the total number of soap and sanitizer dispensing events divided by the total number of opportunities × 100. Importantly, this rate was not the proportion of opportunities associated with a dispensing event, which was the true compliance rate. Although this proportion may be estimated from direct observation (ignoring the Hawthorne effect), the performance rates typically reported from AHHMS are assumed to be an estimate of the compliance rate because opportunities are not correlated to a given event when calculating the performance rate on a specific unit. Statistical analyses in this study were performed by fitting a Poisson general additive mixed model (GAMM) to the monthly performance rates, and a logistic regression GAMM was fit to the monthly ratio of soap to sanitizer dispensing events. Both types of GAMMS included a random effect for unit and a smoother to model nonlinear trends over time. Model assumptions were verified with Pearson residual plots. Wald confidence intervals (CIs) and *P* values were reported to assess contrasts with respect to study phase and unit type. All statistical analyses were performed using R version 4.0.1 software (R Core Team 2020)^12^ with the *mgcy* ^1^ package.^13^

## Results

**Figure 3.**
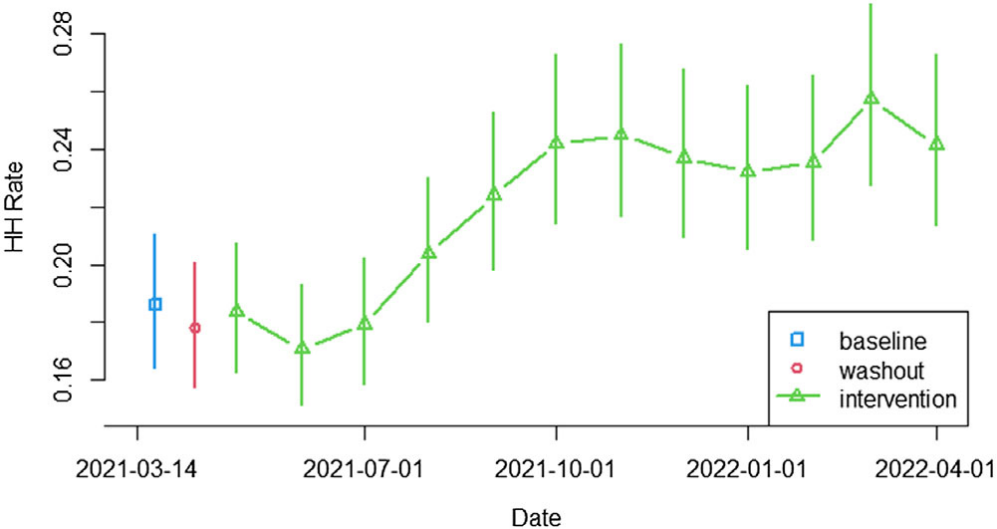
Monthly hand hygiene performance rates for all units. The green curve shows the change in the median hand hygiene rate during the intervention period compared to the baseline and washout periods, with vertical bars showing 95% confidence intervals for the monthly rate.

**Figure 4.**
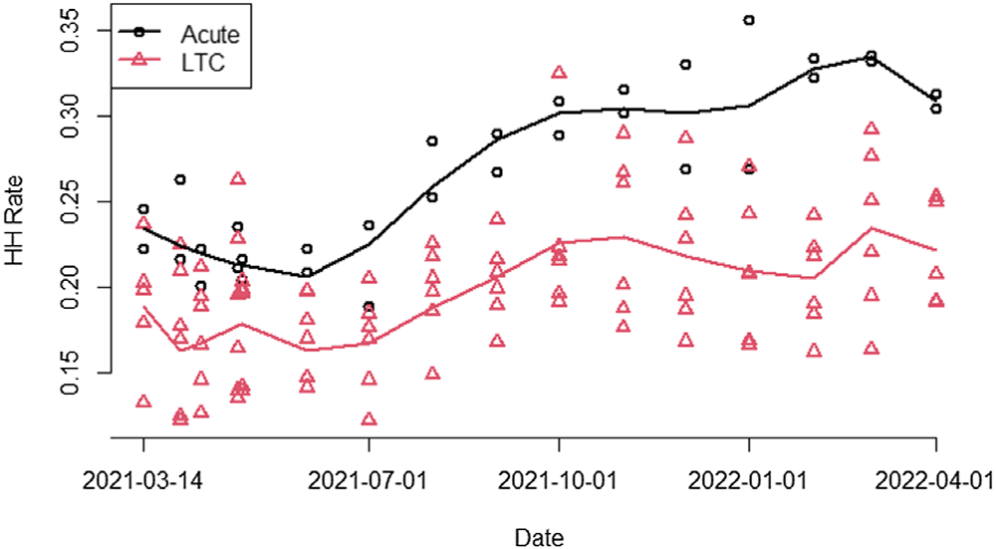
Monthly hand hygiene performance rates for acute care versus long-term care units (the line is the overall median and each data point represents a unit).

During the 4-week baseline period, the median HH performance rate was 18.6 (95% CI, 16.5–21.0) for all 8 units. During the intervention period, the median HH rate increased to 21.6 (95% CI, 19.2–24.4), and during the last 4 weeks of the intervention period (exactly 1 year after baseline) the 8 units exhibited a median HH rate of 25.1 (95% CI, 22.2–28.4; *P* < .0001) (Fig. 3). The median HH rate increased from 17.5 to 20.0 (*P* < .0001) in LTC units and from 22.9 to 27.2 (*P* <.0001) in acute-care units (Fig. 4). HH performance rates for all units are documented in Table 2.

**Table 2.** Summary of HH Performance Details by Unit

Unit	Patient Base	Building	Unit Type	No. of Rooms^b^	Baseline (4 Weeks) (3/14/2021–4/10/2021)			Intervention (∼1 Year) (4/29/2021–4/10/2022)			Postintervention (Last 4 Weeks) (3/14/2022–4/10/2022)		
					Oppor-tunities^c^	Hand-wash Events^d^	ABHR Events^e^	Oppor-tunities^c^	Hand-wash Events^d^	ABHR Events^e^	Oppor-tunities^c^	Hand-wash Events^d^	ABHR Events^e^
1	Acute Care	Tower	MedicalSurgical	24	68,157	5,932	11,080	885,824	73,284	177,492	70,692	6,267	17,218
2	Acute Care	Tower	General Medical	24	72,986	4,710	11,276	1,022,344	50,820	227,309	74,589	3,512	20,741
3	LTC^a^	Tower	SNF	31	77,795	6,252	6,586	937,072	56,688	126,519	81,049	4,069	12,537
4	LTC	Tower	SNF Rehab	13	71,885	5,089	8,356	747,083	64,475	95,944	59,544	5,275	10,245
5	LTC	320	LTC Resident	33	76,674	4,200	5,684	906,181	52,373	97,816	79,575	4,439	11,385
6	LTC	320	LTC Resident	28	50,649	4,826	5,576	647,454	55,566	80,033	51,765	9,836	5,017
7	LTC	320	Memory	24	29,519	3,208	2,123	353,318	42,045	32,180	30,478	2,617	3,669
8	LTC	320	Hospice	20	27,478	2,420	3,873	465,039	33,657	79,862	43,552	2,886	8,552

Note. LTC, long-term care; SNF, skilled nursing facility; rehab, rehabilitation; ABHR, alcohol-based hand rub.

a The VA typically refers to this patient population as veterans within a “community living center.”

b This refers to the number of rooms monitored (not occupancy) on the unit to provide a directional sense of scale.

c The combined number of veteran room entries and exits.

d The number of times a dose of hand soap was dispensed.

e The number of times a dose of hand sanitizer was dispensed.

**Figure 5.**
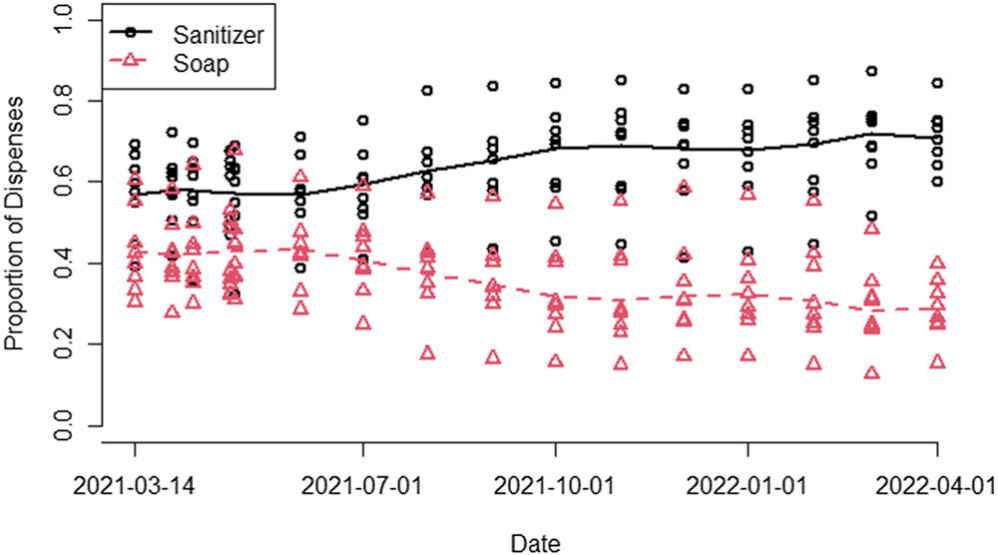
Monthly proportion of sanitizer and soap dispensing events (the line is the overall median and each data point represents a unit).

The intervention and educational efforts to hand wash only when necessary (eg, visibly soiled hands) resulted in an increased use of hand sanitizer from 57.5% of HH events during baseline to 65.1% (*P* < .0001) (Fig. 5), with the ratio of hand sanitizer to hand soap trending up in all units until hand sanitizer dispensing events were 2.4 times greater than soap dispensing events by the end of the study. All units received consistent feedback to improve HH performance rates by both increasing HH events and decreasing HH opportunities: (HH event increase)/(HH opportunity decrease) = increased HH performance rate. Units were encouraged to decrease HH opportunities by bundling care to improve efficiency and productivity and to reduce “artifact” recordings by keeping doors to unoccupied rooms shut to prevent unnecessary entry. The overall increase in HH performance rates was primarily due to HH events increasing in all units. For example, HH events increased from 88,758 dispensing events during the baseline to 123,722 dispensing events during the last 4 weeks of the intervention (see details by unit in Table 2). Direct observation results during the same periods showed HH compliance rates ranging from 61% to 86% (data not shown), which was consistent with direct observation HH compliance results prior to AHHMS installation.

## Discussion

In this study, implementation of an AHHMS combined with multiple complementary strategies increased median HH performance rates on all study units at the Dayton VAMC, with a relative increase of 34.8% over baseline in 1 year. Although the measured performance rate during the study was significantly lower than that obtained by direct observation, prior research has shown that direct observation generally overestimates actual HH performance,^14–16^ so our finding is not surprising.

Maintaining a high profile for HH compliance appeared to be a key component for improved performance. Weekly HH huddles that included infection preventionists, nurse managers, and ancillary staff provided a context in which barriers and successes could be shared promptly. Creative signage was intermittently utilized to serve as an additional reminder of the importance of HH in the prevention of HAIs in our veteran population, as promoted by the “Clean Hands Count” campaign of the Centers for Disease Control and Prevention.^17^ Although special events, such as a grand rounds and a booth for World HH Day provided additional focus, perhaps the most important strategy was the provision of regular feedback regarding HH rates, which is supported by a number of other studies.^18–22^ Consistent improvement in the composite rate across all units was noted following the provision of daily unit-specific rates to nurse managers in week 19 (Fig. 1). Figure 1 provides a timeline for implementation of other complementary strategies and the overall effect on HH performance.

Additional factors that may have contributed to increasing HH performance rates included optimizing the number and placement of ABHR dispensers. The benefit of making adjustments in dispenser placement according to “space syntax” (ie, the analysis of spatial layouts and human activity in buildings to improve efficiency) in a healthcare facility has been shown in a number of studies.^23–26^ Although dispenser placement decisions on our campus primarily occurred prior to the study period, the need for optimization became apparent during the weekly huddles. Indeed, valuable information was obtained by reviewing unit- and room-specific opportunities and events that resulted in additions and adjustments. These included the installation of additional ABHR dispensers in unit hallways, which providers preferred to save time while walking to a specific room. ABHR stands were also placed in busy, open locations such as the resident dining areas on LTC units.

Our study demonstrated an increasing hand sanitizer to soap ratio over time (Fig. 4); others have shown improvement in HH performance with introduction of or improved access to waterless hand sanitizer.^27^ It is unclear whether this observation was due to increased access to any dispenser, educational efforts to highlight ABHR strengths, a preference for waterless hand sanitizer among the staff, or some other factor. The rates of *Clostridioides difficile* colitis were unchanged throughout the study period and did not affect the ratio. Our overall hand sanitizer to soap ratio of 65% is rather low compared to other hospitals in North America.^28^ This may be at least partly due to greater soap use in the LTC units, where soap is still preferred by some HCWs.

Several challenges to maximizing the impact of the AHHMS became apparent during the study. High nursing staff and unit manager turnover necessitated extra oversight and personnel time to achieve prompt, effective training with the system. Indeed, others have described the essential role that nurse unit managers play in fostering the morale and social cohesion that is an important component to successful HH performance on a specific unit.^29^ In addition, consistently effective HH by ancillary staff performing high-frequency visits (eg, foodservice and custodial workers) required multiple conversations over several months. Being sensitive to our approach to staff became necessary when some reacted negatively to “Oh SNAP” and other verbal reminders of appropriate HH use. Lastly, patience was required by everyone involved when the causes of periodic HH rate fluctuations remained elusive. Analysis of HH rates in several studies during the coronavirus disease 2019 (COVID-19) pandemic showed a rapid increase at the onset of the pandemic (March 2020), but a decrease approximately back to prepandemic rates within 3 months thereafter.^30–32^ Multiple hypotheses were proposed, but this finding reinforces how challenging it is to sustain increased HH rates and motivation for improvement. The analogy of running a marathon versus a sprint is frequently mentioned in our weekly HH huddles.

The use of AHHMS in LTC facilities has not been extensively studied, but several challenges and opportunities in this setting have previously been described. Many patients who enter these “postacute” units are already colonized or infected with multidrug-resistant organisms and/or *Clostridioides difficile*. These patients introduce additional risk for infectious outbreaks that may be mitigated when using an AHHMS.^10,33^ Our experience highlighted some unique challenges. For example, the tendency for resident veterans to sit or linger in their doorway (a location they might consider their “front porch”) triggered the opportunity counter for this AHHMS and thereby contributed to the lower HH performance rates in LTC units. The increased mobility and visitor traffic on these units also created more opportunities for hand hygiene, and further study on the use of the AHHMS on LTC units would be beneficial to optimize their use in this setting.

This study had several limitations. It had a nonrandomized design, with implementation of multiple interventions in a stepwise fashion. As a result, we were unable to establish the extent to which each complementary strategy contributed to increased HH performance rates. HAIs were not reported as part of the study, and the relatively small hospital size precluded any definitive deduction in that regard. A 4-week baseline period was suboptimal in comparison to the full-year intervention period, but we felt this to be practical because a longer baseline period would delay the hospital’s HH intervention efforts. Additionally, the AHHMS provided several orders of magnitude more HH opportunities during this period than could be achieved with direct observation. Nevertheless, we attempted to mitigate this issue, as well as account for seasonal effects, by comparing the 4-week baseline to the same 4-week period exactly 1 year later. Similar to direct observation in many cases, the AHHMS is unable to account for all 5 moments of hand hygiene, including whether a body fluid exposure in a patient’s room prompts an additional HH event (HH moment 3). As previously stated, the system is also unable to account for the WHO exception allowing for a single HH event when leaving one patient room and immediately entering another. Furthermore, this study occurred amid the COVID-19 pandemic, which caused limitations to movement of patients and visitors. A comparison of HH rates obtained by direct observation and the AHHMS would be helpful, but a comparison was not powered sufficiency due to the vast difference in sample size between the 2 groups. Finally, a study in adult acute-care hospitals suggested ∼85% of all patient room entries and exits are from HCWs.^32^ However, with this type of AHHMS, there is no way to estimate the number or proportion of HH opportunities that are the result of movement of patients and visitors. This limitation likely contributed to the large discrepancy between the direct HH observations and the performance rate captured by the AHHMS.

In conclusion, complementary strategies bundled into a HH intervention program after the installation of an AHHMS resulted in increased HH performance for all units. Although it is possible that the complementary strategies provided some improvement in HH rates independent of the AHHMS, the AHHMS is a tool that provides more robust data and a higher profile to the challenge of maintaining excellent HH than could be achieved with direct observation alone. Acute-care unit performance was consistently higher than LTC units due to multiple inherent challenges in the subacute setting. Appropriate goals and expectations on LTC units will become clearer as experience with AHHMSs grows, and this is an opportunity for further study. Future HH improvement on all units will rely on continued adaptation of complementary strategies and long-term monitoring. Additional study opportunities include determination of (1) the most effective complementary strategies and (2) whether different types of AHHMSs have an advantage over others in various settings.
